# The innate aptitude’s effect on the surgical task performance: a systematic review

**DOI:** 10.1007/s13304-021-01173-6

**Published:** 2021-09-25

**Authors:** Michael El Boghdady, Beatrice Marianne Ewalds-Kvist

**Affiliations:** 1 Department of General Surgery, Croydon University Hospital, London, UK; 2 St Georges University of London, London, UK; 3 Stockholm University, Stockholm, Sweden; 4 University of Turku, Turku, Finland

**Keywords:** Innate aptitude, Psychomotor skills, Surgical performance, Aptitude for surgery, Manipulative abilities, Laparoscopic surgery

## Abstract

Surgery is known to be a craft profession requiring individuals with specific innate aptitude for manipulative skills, and visuospatial and psychomotor abilities. The present-day selection process of surgical trainees does not include aptitude testing for the psychomotor and manual manipulative skills of candidates for required abilities. We aimed to scrutinize the significance of innate aptitudes in surgical practice and impact of training on skills by systematically reviewing their significance on the surgical task performance. A systematic review was performed in compliance with PRISMA guidelines. An initial search was carried out on PubMed/Medline for English language articles published over 20 years from January 2001 to January 2021. Search strategy and terms to be used included ‘aptitude for surgery’, ‘innate aptitude and surgical skills, ‘manipulative abilities and surgery’, and ‘psychomotor skills and surgery’. MERSQI score was applied to assess the quality of quantitatively researched citations. The results of the present searches provided a total of 1142 studies. Twenty-one studies met the inclusion criteria out of which six citations reached high quality and rejected our three null hypothesis. Consequently, the result specified that all medical students cannot reach proficiency in skills necessary for pursuing a career in surgery; moreover, playing video games and/or musical instruments does not promote skills for surgery, and finally, there may be a valid test with predictive value for novices aspiring for a surgical career. MERSQI mean score was 11.07 (SD = 0.98; range 9.25–12.75). The significant findings indicated that medical students with low innate aptitude cannot reach skills necessary for a competent career in surgery. Training does not compensate for pictorial-skill deficiency, and a skill is needed in laparoscopy. Video-gaming and musical instrument playing did not significantly promote aptitude for microsurgery. The space-relation test has predictive value for a good laparoscopic surgical virtual-reality performance. The selection process for candidates suitable for a career in surgery requests performance in a simulated surgical environment.

## Background

Traditionally, the selection of surgical trainees has mainly been based on academic achievements and subjective assessments obtained from non-structured interviews [1, 2]. While surgery is known to be a craft profession requiring individuals with specific innate aptitude for manipulative skills, the present-day selection process of surgical trainees in most countries does not include aptitude testing for the visuospatial, psychomotor, and manual manipulative skills of candidates. Consequently, we focused on innate aptitudes in the selection process of suitable candidates for surgery and we defined ‘innate aptitude’ as an ‘inborn or congenital skill, talent, or inclination to perform and complete a task with or without training.

Some researchers quantified the size of medical students’ innate psychomotor skills and recommended to recruit technically gifted candidates for surgery [3], while others concluded that laparoscopy aptitude tests constitute valuable additions to the assessment of candidates for medical specialties that require laparoscopic skills [4].

We aimed to scrutinize the significance of innate aptitude in surgical practice and training by systematically reviewing its significance on the surgical task performance. We also reviewed innate aptitude along with other non-surgical skills, for example, playing video games or musical instruments, while skills in these non-domain-specific areas have been thought to facilitate the learning of aptitudes needed for surgical task performance. In addition, the predictors for innate aptitude for surgical skills were reviewed.

## Methods

### Protocol

A systematic review was performed in compliance with the PRISMA (Preferred Reporting Items for Systematic Review and Meta-Analysis) guidelines [5].

### Search strategy and criteria

A search covering 20 years was carried out by means of PubMed/Medline for English language articles published from January 2001 to January 2021. Search strategy and terms to be used included ‘aptitude for surgery’, ‘innate aptitude and surgical skills, ‘manipulative abilities and surgery’, and ‘psychomotor skills and surgery’.

Only publications related to innate aptitude for surgery were included across all surgical specialties with the exception of veterinary surgery. Surgical tasks in surgical training programs and surgical performance in experimental surgical studies were included. Based on the requirements of the Medical Education Research Study Quality Instrument (MERSQI) to assess the quality of citations, quantitatively researched citations were included [6]. Qualitative papers, reviews, conference abstracts, letters, editorials and commentaries, protocols, and non-English publications were excluded.

The retrieved citations were read in full text for further assessment for eligibility. Risk of bias was assessed across studies.

### Quality measurement

The Medical Education Research Study Quality Instrument (MERSQI) was applied to assess the quality of quantitatively researched citations [6]. The MERSQI contains ten items that reflect six domains of study quality including study design, sampling, type of data, validity of evaluation instrument, data analysis, and outcomes. The maximum score for each domain was three with a potential range from 5 to 18. The MERSQI score represents the mean of two independent assessors’ quality estimations of each citation. For the currently included 21 citations, the quality mean was 11.07 (SD = 0.98) scores and the scores ranged from 9.25 to 12.75. Scores < 9 would have revealed insufficient quality but were not present. Scores from 9 to 10.25 represented low quality, scores from 10.50 to 11.75 revealed moderate quality, and ≤ 12 scores indicated high quality. The distribution of aptitude scores was found to be normal when computed by one-sample Kolmogorov–Smirnov test (two-tailed *p* = 0.062 > 0.05; Lilliefors corrected).

## Results

### Study selection and characteristics

**Fig. 1:**
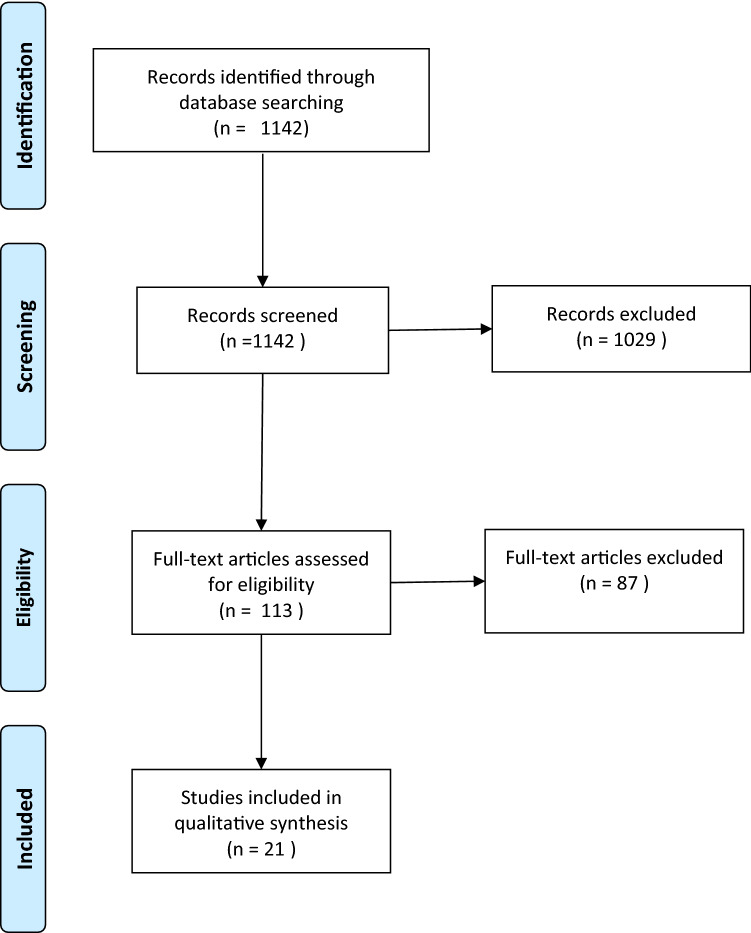
Flow diagram

The results of the present searches over 20 years provided a total of 1142 studies. These studies were screened and assessed for eligibility. After the inspection of the titles and abstracts, these elements were systematically reviewed against the inclusion and exclusion criteria, and 113 papers were retrieved out of which 87 papers were excluded (Fig. 1). After administration of inclusion and exclusion criteria, 21 articles remained in this review. Search items were studied from the nature of the article, date of publication, forum of publication, aim and main findings in relation to the effect of innate aptitude on the surgical performance, as well as quality scores in agreement with the MERSQI protocol.

### Results of individual studies

The definitions and tests of various forms of innate aptitudes are shown in Table 1. Tabular analysis of the revealed citations is presented in Table 2. Out of the 21 citations, 11 benefited from virtual reality simulators (VRS) and ten from dry lab. No significant difference between quality scores was found between these two groups VRS M 10.98 [SD 0.96] vs. M 11.20 [SD 0.99]; *p* = n.s.). Medical students and surgical novices participated in 18 citations, trainees in three citations, and they were compared to expert surgeons in four citations.

**Table 1:** Innate aptitude’s definitions and tests taking place in the present review

Innate aptitude	References	Definition and testing
Depth perception	Cowie (1998) [7]	Depth perception refers to the visual ability to perceive the world in three dimensions and to evaluate the distance of an object. Pictorial surface orientation testing was developed to assess a subject’s perceptual ability in laparoscopic surgery
Perceptual skill	Cowie (1998) [7]	Perceptual skill denotes the ability to envisage recovering information about 3-D structures from 2-D monitor displays to assess its impact on surgical performance. Space-relation test was constructed to test this ability
Psychomotor aptitude	Dikmen et al. (1999) [8]	Psychomotor aptitude means performing motor tasks with exactitude and dexterity. For example, using manual and finger dexterity and hand–eye coordination. This was assessed using a grooved pegboard
Space-relation ability	DAT (1996) [9]	To assess the ability to visualize a three-dimensional object from a two-dimensional pattern and to visualize how this object would look if rotated in space, the space-relation test (SRT) was constructed
Visual–spatial aptitude	Carrol (1993) [10]	Visual–spatial aptitude is the ability to generate, transform, and retain structured visual images. That is, to mentally manipulate two-dimensional and three-dimensional figures

**Table 2:** Tabular analysis of the included citations

Author (year)	Journal	Objective	Findings	MERSQI^a^	Quality
Francis et al. (2001) [11]	The American Journal of Surgery	To determine endoscopic consultant surgeons’ level of three aptitude tests: the Gibson Spiral Maze Test (eye-hand coordination), the Crawford Small Parts Dexterity Test (manual dexterity), and the Space Relations Test (visuospatial ability) compared with those of medical students’ and the reference norm as provided by the tests’ manuals	The level of eye-hand coordination and manual dexterity of master surgeons was found to be higher vs medical students, while master surgeons visuospatial ability was lower	12	High
Dashfield et al. (2001) [12]	The Royal College of Surgeons of England	To study correlation between the scores achieved on a computerized psychometric test, assessing psychomotor aptitude, and learning tying of a surgical reef knot. A fully automated selection system, MICROPAT = micro-computerized personnel aptitude tester, assessing both psychomotor and information management ability, was developed and validated. MICROPAT correlates with psychomotor tests and nasotracheal endoscopy as well as with performance of obstetric extradural anesthesia in anesthetic trainees while learning these new skills	Although it is not essential for a trainee to undergo psychomotor testing before boarding on a surgical training program, there may be some advantages for the trainee’s future career in having an objective evaluation of the individual's psychomotor aptitude An awareness of trainees' psychomotor abilities may help instructors to tailor training programs more effectively for the individual	10.25	Low
Gallagher et al. (2003) [13]	Surgical Endoscopy	To assess in trainees pursuing a career in surgery empirically the relationship between laparoscopic ability and the perceptual skill of recovering information about 3-D structures from 2-D monitor displays	Relevant battery of tests could and should include measures of perceptual skills related to recovering information about depth from pictures. PicSOr provides a straightforward measure of ability in that area. It offers a possible mean of assessing which trainees have the fundamental visuospatial and depth perception abilities to ‘‘potentially’’ learn laparoscopic surgery (i.e., natural aptitude). The next step in validating PicSOr is to demonstrate that it predicts laparoscopic skill in the operating room	12	High
Schijven et al. (2004) [14]	Journal of Surgical Report	To link surgical novices’ psychometric ability to a test battery data with actual accomplishment result on an objective, validated, and reproducible surgical laparoscopic task using virtual-reality simulation. Subjects were examined using the aptitude test battery including the Abstract Reasoning test (ART), the Space Relations test (SRT), the Gibson Spiral Maze test, and the Crawford Small Parts Dexterity tester	The ART and SRT had a predictive and selective value in identifying people who achieve good laparoscopic surgical execution on the Xitact simulator. ART is the solitary aptitude test which correlates directly with Xitact performance. The test itself is highly correlated to SRT. Thus, the concurrent validity of the Xitact LS500 with the combination of the SRT and ART measuring an individual’s visuospatial abilities was proven	12	High
Hislop et al. (2006) [15]	Journal of Vascular Surgery	To scrutinize definition and measurement of innate endovascular aptitude and empirically correct performance to verify if these are two separate things by a modification of a previously validated scale Modified Reznick Scale (MRS)	Innate endovascular aptitude and empirically correct performance can be two separate things, and aptitude is perhaps acquirable through (or identified by) vast nonmedical video game experience. The MRS correlates with endovascular experience and formal training. Experts and novices with broad video game experience get short completion times, whereas high MRS scores are attained only by formally trained subjects	11.25	Moderate
Rosenthal et al. (2006) [16]	Surgical Endoscopy	To test that subjects show logarithmic performance curves on repetitive trials for a component task of laparoscopic cholecystectomy on a virtual-reality simulator (VR), and that interindividual differences in performance after considerable training are significant	Assessment of perceptual motor skills and the innate ability of an individual with no prior experience in laparoscopic surgery to develop such skills using the LS 500 VR simulator is feasible and reliable. The training of perceptual motor skills and rating of innate ability for the growth of such skills is feasible and reliable. Inter-individual differences can be rated with performance measurements over only a few repetitive trials	10.5	Moderate
Cope and Fenton-Lee (2008) [17]	ANZ J Surg	To assess the innate psychomotor skills of interns and to compare those drawn to a surgical career with those with no interest in a surgical career	The Minimal Invasive Surgical Trainer, Virtual Reality (Mentice) Simulator provides an objective and similar evaluation of laparoscopic psychomotor skills. Interns with and without inherent abilities aspire to pursue surgical careers and their aptitude does not influence this decision. Surgical colleges could use psychomotor ability assessments to recruit candidates to pursue a career in surgery. Trainees needing closer monitoring and additional training could be identified early and guided to achieve competency	9.25	Low
Nomura et al. (2008) [18]	Journal of Surgical Education	To explore the predictive factors that affect laparoscopic skills by evaluating medical students’ laparoscopic simulator training for outcome data. The 43 participants performed an object-positioning module on the ProMIS simulator. Execution time, instrument path length, and economy of movement for each trial were recorded	The participants’ interest in laparoscopic surgery and success in playing the piano did not affect the ability in any of the three assessment measures of the simulator. The students who were interested in TV games completed the task in less time and had a shorter left instrument path length. Those who were confident about driving and considered themselves manually dexterous completed the task in less time The aptitude was revealed by interviewing the participants beforehand about attraction to TV games, manual dexterity, and confidence about driving. TV gaming and driving require the same abilities as laparoscopic surgery. While, psycho- motor, perceptual, or visuospatial ability are crucial for good performance	10.50	Moderate
Van Herzeele et al. (2010) [19]	Journal of Vascular Surgery	Endovascular virtual-reality simulation of medical students was performed to answer the question if innate perceptual, visuospatial, and psychomotor aptitude (VSA) can project levels of endovascular skills	Simulator-based training in endovascular skills improved performance in medical students. Initial endovascular aptitude and fine motor dexterity as well as image recall at end of the training period, correlated. For recruitment approaches, VSA can be used for predictive validity studies	11.25	Moderate
Alvand et al. (2011) [20]	The Journal of Bone and Joint Surgery	To investigate the innate arthroscopic skills and learning curve patterns of medical students	Variation in innate arthroscopic skill exists among future surgeons, with some individuals being unable to achieve competence in basic arthroscopic tasks despite sustained practice. Identifying individuals who lack innate arthroscopic skills early in their career, in order to provide them with focused training and relevant career guidance, may be of great value	11.0	Moderate
Kennedy et al. (2011) [21]	Journal of Surgical Education	To find out if regularly playing video games forecasts psychomotor performance on a laparoscopic simulator or scores on tests of visuospatial and perceptual abilities, and to examine the eventual correlation between these innate abilities	Regular video gaming correlates positively with psychomotor ability, but it does not seem to influence visuospatial or perceptual ability. Some students may have better psychomotor abilities that increase their interest in activities involving manual dexterity. Video game experience might be beneficial to a future career in surgery and relevant surgical skills may be gained usefully outside the operating room in activities that are not related to surgery	9.25	Low
Nugent et al. (2012) [22]	International Journal of Colorectal Disease	To determine if fundamental aptitude impacts on ability to perform a laparoscopic colectomy	There is a relationship between aptitude and ability to perform both basic laparoscopic tasks and laparoscopic colectomy on a simulator. The findings suggest that there may be a role for the consideration of an individual’s inherent baseline ability when trying to design and optimize technical teaching curricula for advanced laparoscopic procedures	9.75	Low
Buckley et al. (2013) [23]	Journal of American College of Surgeons	To assess whether aptitude has an impact on ability to achieve proficiency in completing a simulated minimally invasive surgical procedure	Candidates with high innate ability became proficient at completing a laparoscopic appendectomy at a faster rate than those with lesser innate ability. The data supported for an objective multifaceted selection process to select suitable trainees for future training programs	10.50	Moderate
Buckley et al. (2014) [24]	The American Journal of Surgery	To compare the rate at which 2 groups of surgical novices became proficient in laparoscopic suturing and intracorporeal knot tying. These 2 groups were at opposite ends of the aptitude spectrum	High aptitude predicts a faster learning curve and improved performance in laparoscopic suturing. A significant number of candidates with low innate ability are unable to reach proficiency despite repeated practice. This study supports the concept of using objective selection processes based on aptitude to select suitable trainees who are likely to prosper in the challenging field of surgery if selected	12	High
Moglia et al. (2014) [25]	Surgical Endoscopy	To test aptitude for psychomotor and manipulative skills of candidates for surgery	In terms of innate aptitude for manipulative and psychomotor abilities, the current study has documented two subpopulations that fall outside the norm for the group of medical students recruited for the stud (1) a small group (6.6%) with a high level and (2) and (3) larger cohort (11.6%) with low level (significantly below the norm) innate aptitude for surgery. Exposure to video game experience did not appear to influence performances on the da Vinci Skills Simulator	11.75	Moderate
Osborn et al. (2015) [26]	Otology and Neur-otology	To determine whether a past of video gaming or musical instrument playing would predict aptitude for a microsurgical task	Musicians who began playing at a younger age did better than those who began playing later; yet, non-musicians performed at a similar level to the best of the musicians. So, it was difficult to draw any conclusions about the potential impact of early musical training. No improvement in microsurgical aptitude was seen in subjects who had a history of video gaming or musical instrument playing	12	High
Moore et al. (2015) [27]	The Laryngo-scope	To evaluate the correlation between the results of a surgical aptitude test administered to otolaryngology residency/training applicants and their performance during training	The residents’ 24 consecutive composite and attitudinal scores were analyzed for correlation with residency performance score by regression analysis. The residents were evaluated for overall quality as a clinician by eight faculty members who were blinded to the results of surgical aptitude testing. The results of these assessments showed good inter-rater reliability. Both the overall aptitude test scores and the subset attitudinal score showed reliability in predicting performance during training	11.75	Moderate
Groenier et al. (2015) [28]	Journal of Surgical Education	To examine the impact of surgeon’s cognitive and psychomotor ability on the training duration and learning rate of performing minimally invasive surgery	Perceptual speed (PS) predicted training duration. Cognitive and psychomotor ability predicted the learning rate of time to accomplish the task. The learning rate for partakers with higher levels of PS was quicker. Psychomotor ability also projected the learning rate for damage to tissue and efficiency of movement. Partakers with better psychomotor ability surpassed other partakers across all sessions on all result estimations	10.5	Moderate
Siska et al. (2015) [29]	Journal of Surgical Education	To answer the following questions: What is the relative contribution of the variables: innate ability vs motivation and voluntary practice in the acquisition of basic laparoscopic skills? To what extent are these baseline variables related?	Medical students’ psychomotor aptitude and motivated practice equally influence final box trainer’s performance. Students with lower aptitude do not automatically train more. Although the interest in surgery was initially not related to psychomotor aptitude, eventually students with high aptitude apply more frequently for a surgical career	11.75	Moderate
Moglia et al. (2018) [30]	Surgical Endoscopy	To quantify the size of individuals with high, average, and low level of innate psychomotor skills among medical students	This study identified two outlying groups: one gifted with a high-level innate aptitude for manipulative skills comprising 5.8% of the total cohort, and a larger group accounting for 11.0% with a lower innate aptitude than their peers. This study supports the growing view among directors and Heads of Surgical Departments that objective testing in a validated surgical VR simulator should be included to complement the selection process for surgical training programs	12.75	High
Mitchell et al. (2019) [31]	Journal of Surgical Education	To assess psychomotor aptitude of medical students drawn to pursuing a procedural career as well as to explore the relationship between real and perceived aptitude and finding predictors of superior aptitude	Students drawn to a procedural career exhibited better psychomotor aptitude, and most of these students found themselves having above-average aptitude compared to peers. Yet, > 25% of all students, regardless of career interest, wrongly over- or under-assessed their psychomotor aptitude. Laparoscopic simulation itself may be valuable to detect and help students with an interest in a procedural field but with a low psychomotor aptitude. These students profit from directed, early interventions, to help them make correct career decisions and increase performance among candidates interested in procedural fields	10.5	Moderate

^a^10.09–12.05 (for 68%) = 10–11.75, moderate 9–10.25 = Low; 10.50–11.75 = moderate; ≥ 12 = High

Out of the 21 citations, six reached high quality through MERSQI ratings and our three null hypotheses were rejected based on the findings from these high-quality citations (Table 3) which significantly overridden the other citations. The difference between high-quality papers and medium/low quality was significant (*M* 12.13 [SD 0.32] > *M* 16.65 [SD 0.82], *t* [19] = 4.221, *p* < 0.0005 two-tailed).

**Table 3:** Citations with high MERSQI scores and null hypotheses

Research	Null hypotheses	MERSQI
Citations	Regardless of level of innate aptitude, all medical students can reach proficiency in skills necessary for pursuing a career in surgery	Quality scores
Buckley et al. (2014) [24]	*Findings:* Some low-aptitude medical students cannot reach proficiency in skills necessary for pursuing a career in surgery	High 12.0
Gallagher et al. (2003) [13]	Training does not compensate for deficiency in skills needed for laparoscopy	High 12.0
Citations	Playing video games and/or musical instruments promote skills for microscopic surgery through transfer	
Moglia et al. (2018) [30]	*Findings:* No significant Spearman correlation with video games and/or musical instruments were found in medical students with high, average, and low level of innate psychomotor skills	High 12.75
Osborn et al. (2015) [26]	No disparity in microsurgical aptitude between gamers and non-gamers was found. Musicians who began playing at a younger age did better than those who began playing later; yet non-musicians performed at a similar level to the best of the musicians	High 12.0
Citations	There are no test with predictive value for novices aspiring for a surgical career	
Francis et al. (2001) [11]	*Findings: The space-relation test (SRT)* has predictive and selective value, identifying individuals who have good laparoscopic surgical virtual-reality performance, given that the age norm is followed. The norm is based on an analysis of 236 adults (mean age 30.25 years) receiving career guidance in the United States with varied educational background	High 12.0
Schijven et al. (2004) [14]	The *space-relation test (SRT)* has predictive and selective value, identifying individuals who have good laparoscopic surgical virtual-reality performance	High 12.0

#### The first null hypothesis

‘Regardless of level of innate aptitude, all medical students can reach proficiency in skills necessary for pursuing a career in surgery’ was rejected, while aptitude plays a key role in learning advanced skills necessary for laparoscopy [13, 24]. Namely, Buckley et al. tested innate aptitude by scoring surgical novices. Group A (*n* = 10) included students with a high level of innate aptitude and another group B (*n* = 10) comprised students with low level of innate aptitude in visual spatiality, depth perception, and psychomotor skills [24]. The results indicated that group A with novices with high aptitude achieved proficiency after a mean of seven attempts (range 4–10). In group B with novices with low aptitude altogether 30% achieved proficiency after a mean of 14 attempts (range 10–16). In group B, 40% demonstrated improvement but did not attain proficiency, and a total of 30% failed to progress. In other words, aptitude plays a significant role in learning advanced skills as candidates with low innate ability were unable to reach proficiency despite repeated training.

Gallagher et al. suggested that perceptual skills connected to recovery of data about depth from pictures are applicable to changes in laparoscopic routine [13]. The ability to recover depth from pictures develops progressively during childhood, and the skill improves with operating laparoscopically. While no differences between experienced surgeons and other groups regarding PicSOr were observed, it seems probable that the skill is fixed in adulthood. Therefore, preselection for pictorial skills is reasonable, while training does not compensate for pictorial-skill deficiency. Gallagher et al. recommend that a relevant battery of tests could and should include measures of perceptual skills related to recovering information about depth from pictures. PicSOr provides a simple measure of talent in that area. As such, it offers a possible way of assessing which trainees have the fundamental visuospatial and depth perception aptitudes to eventually learn Minimal Access Surgery (i.e., natural aptitude). Yet, both Buckley et al. and Gallagher et al. supported the use of objective selection processes based on innate aptitude to choose suitable trainees who are likely to succeed in surgery, if selected [13, 24].

#### The second null hypothesis

Playing video games and/or musical instruments promote skills for microscopic surgery through transfer from gaming and playing instruments. The null hypothesis was rejected based on findings in two high-quality studies [26, 30].

Osborn et al. determined whether video gaming or musical instrument playing would predict aptitude for a microsurgical task [26]. Altogether 46 students performed a microsurgical task using a novel simulator and their performance was assessed by blinded raters. There was no correlation between video gaming and improved microsurgical performance. Most students played a musical instrument. Within this group, the highest scores were obtained by musicians who began playing before the age of 6. However, musicians did not obtain higher scores than non-musicians, regardless of their age of commencement. Thus, no improvement in microsurgical aptitude was seen neither in students who had a history of video gaming nor from musical instrument playing.

Moglia et al. studied a volunteer sample of 155 medical students and the large majority (83.2%) of the participants was found to possess average aptitude for surgery [30]. Out of nine top performers, five had experienced both video-gaming and musical instrument playing, but Spearman rho correlation both with video-gaming and musical instrument playing was non-significant. Seventeen students underperformed, eight with skills in video games and musical instrument, three with experience in video-gaming; and five with musical instrument playing but Spearman rho correlation with video games and musical instruments was non-significant. Also, the 129 subjects with average performances had no significant Spearman rho neither with video games nor with musical instruments.

#### The third null hypothesis

There are no tests with predictive value for novices aspiring for a surgical career. The null hypothesis was rejected based on findings by Francis et al. and Schijven et al. [11, 14]. Francis et al. administered The Space Relations Test (SRT) to 20 master endoscopic surgeons (age median 47; range 38–57) compared to 20 medical students (age median 21; range 18–28) with no previous experience in surgery [11]. The researchers found that if the age norm for this test is followed, SRT is a valuable test for surgical aspirants. Francis et al. claimed that because of a possible age factor effect, it would be unwise to dismiss tests of visuospatial ability in aptitude assessments for selection of surgical trainees. The test’s age norm is based on an analysis of 236 adults (mean age 30.25 years) receiving career guidance in the United States with varied educational background. SRT is a subtest of the Technical Abilities Battery of the Differential Aptitude Tests (Psychological Corporation Ltd.). It evaluates the aptitude to reconstruct a three-dimensional object from a two-dimensional pattern as well as the ability to conceptualize the object when rotated in space. The test consists of 50 tasks. For each task, there is one unfolded test diagram and four optional folded patterns, one of which results in the correct folded diagram. The novice aspiring for a surgical career must identify the appropriate alternative [9].

Schijven et al. [14] found that SRT had a predictive and selective value in identifying people who achieve good laparoscopic surgical act on the Xitact simulator. SRT is highly correlated with The Abstract Reasoning Test (ART) [11]. Partakers scoring > 45 on the SRT and > 35 on ART are unlikely to be unsuccessful on the Xitact simulator. Yet, a “good” surgeon is more than accumulated knowledge and psychomotor abilities. Personality traits, i.e., interest, endurance, empathy, stress-resistance, and decision-making abilities, are necessary for becoming a good surgeon.

### Risk of bias within and across studies

The Medical Education Research Study Quality Instrument (MERSQI) was designed to evaluate the methodological quality of medical education research [6]. While the MERSQI has been demonstrated to be a reliable and valid instrument for measuring methodological quality in medical education research, we applied it in our systematic review. The risk of bias within studies consisted of the small groups of participants, which fact always is accompanied with bigger SD making it more difficult to get significant results. It was claimed that MERSQI is not limited to intervention studies only, but is appropriate for all quantitative studies [32]. This constitutes the risk of bias across the present study as we had to exclude qualitative and review studies. The MERSQI scale was found to be somewhat robust and clumsy as well as demanded discussions between raters for the final inter-rater consensus about citations’ quality scores. The maximum scores of MERSQI was 18, but no citation came close to that number.

## Discussion

We studied the effect of innate aptitude on the surgical performance. High-quality studies supported the idea that the selection of medical students to a career in surgery should be based not only on academic achievements and subjective assessments such as non-structured interviews [1, 2] but on objective measures considering the surgery’s craft-natured profession requiring individuals with specific innate aptitude for visuospatial and for manipulative skills, not only suitable for open surgery, but more so for sophisticated laparoscopic task performance.

Innate aptitude plays a major role in learning skills necessary for laparoscopy [13, 24]. High innate aptitude for surgery results in better outcomes in operating theaters. It was previously revealed that the most encouraging skills correlating with laparoscopic and arthroscopic simulator success were those of visuospatial aptitude, psychomotor skills, and perceptual talent [33]. All medical students cannot reach proficiency in some of the skills relevant for laparoscopy [34]. On the other hand, it has also been disclosed that aptitude tests can be used to predict parts of the individual differences in laparoscopic skills in forms of useful additions to simulator-based assessment [35]. A laparoscopy aptitude test could help low-aptitude students to make their right career decision. They can invest their time and energy in a specialty that more closely matches their talent.

The idea that video gaming and/or playing musical instruments would promote skills for microscopic surgery did not get support [25, 26]. Tetris is one of the leading video games in the world and has sold in more than 202 million copies. Tetris requires intelligence and skill and was speculated to benefit laparoscopic performance, although video-gaming is a non-domain aptitude. In contrast, domain-specific competence depends on genes, environment, practice, and traits in complex interactions [36]. Two previous experiments explored if expertise on Tetris transfers to measures of spatial ability [37]. Tetris-player experts surpassed non‐Tetris players on mental rotation of shapes if the shapes were identical to or almost similar to those of Tetris, but did not benefit other tests of spatial ability. All in all, the results suggested that spatial expertise is highly domain‐specific and does not transfer broadly to other domains [36].

Also, transfer from playing music to laparoscopic task performances in surgical novices does not occur [36]. The researchers studied the association between music practice and accuracy of motor timing and discovered that the relationship disappeared when controlling for genetics and shared environment. This agrees with a twin study on training of the rotary pursuit task, in which the genetic influences on performance as well as on rate of learning were disclosed [38]. In other words, the selection of medical students to a career in surgery should neither rely on student’s experience of video games nor on their experience of playing musical instruments.

It was claimed that no single test has been reported to reliably predict technical performance across the range of techniques and skills required of surgical trainees [39]. Nevertheless, visual spatial tests have demonstrated some promise, but only in predicting performance on a specific subset of surgical tasks. The SRT was used to assess visuospatial ability and can be used in aptitude assessment for selection of surgical trainees [11]. The importance of visuospatial ability in surgery has been emphasized and it has been suggested that visuospatial ability is related to competency and quality of results in complex surgery, and could be used in resident selection, career counseling, and training [40]. Furthermore, the assessment of visuospatial ability and the use of SRT for selection of surgical trainees have been previously well supported [14]. SRT has been administered to 1391 aspirants to a dental school in a study. Little correlation was found between SRT and GCE 'A' level grades, showing that SRT measured different aptitude but disclosed a strong relationship between students scoring poorly on SRT and then resigning from the course or failing to graduate on time [41].

Traditionally, academic achievement is a strong predictor of successful completion of training programs and success in end-of-training examinations, but does not predict clinical performance during the training program [42]. Therefore, that kind of achievement can be complemented with aptitude tests [33]. Nevertheless, motivation, perseverance, and purposeful practice were considered as greater determinants of technical performance than a score on an aptitude test [35]. Yet, the importance of testing non-technical skills for surgeons by NOTSS was stressed, but it has been claimed that the best option to select candidates suitable for a career in surgery would be assessments in a simulated surgical environment, where the artificial environment replicates the reality, and where the candidates’ skills in forms of teamwork, communication, and response to stress are assessable [43]. However, a simulated operating theater is not achievable in every hospital, and therefore, NOTTS could be used and applied for an early evaluation and training of non-technical skills for medical students aspiring to become surgeons.

### Clinical implication and future direction

The role of psychological motivation for surgical activity is underestimated. The psychological motivation for a career in medical specialties of 318 medical students was studied [44]. Aspects influencing the Specialty preference (SP) were reduced by Principal Component Analysis to the components: 'working situation' (comprising extrinsic motivation), 'specialty prospect' (containing intrinsic motivation), and 'career opportunity' (including dual motivation). Males’ common SP’s were surgical specialties; females interested in surgical specialties were more career driven (i.e., liked prestige, research opportunity, career prospects, and got encouragement from family and professor). Males pursuing surgical specialties, scored higher on 'career opportunity', and they were both intrinsically and extrinsically motivated for their SP. Extrinsically motivated students looked for external sources of support, they needed external rewards for hard work. Yet, medical students were found to over- or under-estimate their factual ability in subjective self-ratings. This fact made us doubt that psychological motivation is enough to successfully pursue a career in surgery which requests complex technical skills. The role of psychological motivation for surgical activity can be the subject for future studies.

The effectiveness of admission interviews has been evaluated as a tool for predicting candidates' performance in the medical program, and it was found in the regression analysis that, for example, in year 2, that the interview scores explained only 3.9% of the variance in the performance in the selection process The selection tools altogether explained 13.5% of that variance and the tools did not correlate with each other. The result was explained in terms that the selection tools worked well alone, but they did not correlate highly with each other, or the interview process did not measure what it was supposed to measure [45].

Therefore, there is a need to evaluate the effectiveness of admission tools against a broad range of outcomes within and beyond the medical program [46]. Our review provided useful examples of selection tools for the measurement of students’ different aptitudes by testing innate aptitudes disclosing suitability for a competent career in surgery.

## Conclusion

The significant findings indicated that medical students with low innate aptitude are not able to reach skills necessary for a competent career in surgery. Training does not compensate for pictorial-skill deficiency, and a skill is needed in laparoscopy. Furthermore, spatial ability does not benefit from video gaming. Also, playing music instruments does not benefit aptitude for microscopic surgery. The mentioned talents are domain-specific competences contingent on genetic factors, environmental essentials, practice, and personality traits. These aspects interact with each other in a multifaceted way. Therefore, the selection of candidates suitable for a career in surgery should ideally be assessed in a simulated surgical environment.
